# Folate: An Emerging Target in Type 2 Diabetes Mellitus and Its Complications

**DOI:** 10.3390/metabo16090685

**Published:** 2026-09-17

**Authors:** Xiaoyu Xiao, Siyi Qin, Zijian Jiang, Yuhui Huo, Junlin Li, Minlan Yang

**Affiliations:** School of Medicine, Yangtze University, Jingzhou 434020, China

**Keywords:** folate, T2DM, diabetic complications, homocysteine, insulin resistance

## Abstract

Folate, which is also known as folic acid, vitamin B9, is a key coenzyme in one-carbon metabolism (OCM) and participates in nucleic acid synthesis, methylation, and redox regulation. In recent years, numerous studies have demonstrated its close association with type 2 diabetes mellitus (T2DM), gestational diabetes mellitus (GDM), and diabetic complications. Serum folate levels are decreased in patients with T2DM, which is associated with increased urinary excretion and metformin-mediated inhibition of folate absorption. The onset of GDM is characterized by a folate dose-dependent imbalance characteristic, high folate combined with vitamin B12 deficiency significantly increases the risk of GDM. Maternal folate status can also modulate offspring insulin sensitivity through epigenetic modifications. The mechanisms by which folate regulates diabetes mainly include lowering homocysteine (Hcy) concentrations, suppressing oxidative stress and inflammatory responses, modulating OCM, and mediating DNA methylation modifications. Cumulative evidence-based data indicate that appropriate folic acid supplementation can improve insulin resistance, restore vascular endothelial function, reduce the risk of cardiovascular diseases (CVDs), diabetic peripheral neuropathy, retinopathy, cognitive impairment, and other complications, and alleviate damage induced by high glucose. This review systematically summarizes the roles and molecular mechanisms of folate in diabetes and its complications, highlighting folate as a potential intervention target for diabetes.

## 1. Introduction

Type 2 diabetes mellitus (T2DM) is a prevalent chronic metabolic disorder predominantly driven by insulin resistance and impaired insulin secretion. In the early stage of the disease, insulin sensitivity decreases in insulin-dependent tissues, including skeletal muscle, adipose tissue, and the liver, which substantially blunts the glucose-lowering actions of insulin. To compensate for the reduced insulin efficiency, the pancreas secretes excessive insulin, resulting in compensatory hyperinsulinemia. Meanwhile, T2DM predisposes patients to a wide spectrum of complications. Microvascular complications primarily consist of diabetic nephropathy, diabetic retinopathy, and peripheral neuropathy, which may progress to renal failure, blindness, and sensory disturbance.

Folate, a genus of vitamin B9 (whereas folic acid is a synthetic form of folate commonly used in dietary supplements and fortified foods) is necessary for cell growth and proliferation in humans, as well as sulfur-containing amino acid metabolism. Dietary folate or exogenous folic acid is metabolized in vivo to produce tetrahydrofolate (THF). THF is further converted into 5,10-methylenetetrahydrofolate (5,10-methylene-THF), which is reduced to 5-methyltetrahydrofolate (5-MTHF), the predominant biologically active form of circulating folate in the human body, under the catalysis of 5,10-methylenetetrahydrofolate reductase (MTHFR). Folate metabolism participates in the synthesis and interconversion of purine and pyrimidine nucleotides. It is generally believed that folate exerts no direct effect on blood glucose levels. However, recent studies have increasingly revealed that folate can significantly ameliorate glycemic profiles and insulin resistance in patients with T2DM, and folate confer benefits for diabetes prevention and the management of diabetic peripheral neuropathy [1,2]. This indicates that folate may become a promising therapeutic target for T2DM and its complications. Accordingly, deeper mechanistic insights into folate in the pathogenesis of T2DM and its complications may help to identify novel therapeutic strategies. In this review, we discuss the biological roles and underlying mechanisms of folate in T2DM and its complications.

## 2. Folate in T2DM and Diabetic Complications

### 2.1. Folate in T2DM

Clinical studies have demonstrated that folate malnutrition is closely associated with the progression of T2DM, and diabetic patients generally present low levels of water soluble vitamins. One study enrolling 100 Omani adults (including 50 newly diagnosed patients with T2DM and 50 age- and sex-matched healthy controls) also found that patients with T2DM commonly had low serum folate and vitamin B12 levels, as well as hyperhomocysteinemia (HHcy) [3]. Serum folate levels are significantly reduced in patients with T2DM [4], yet the underlying causes remain incompletely clarified. In the blood and liver of a streptozotocin (STZ)-induced diabetic rat model, the concentration of water-soluble vitamins remained essentially unchanged, while the excretion of water-soluble vitamins in urine was elevated [5], which indicates that the reduced serum folate levels in T2DM patients may be due to increased folate urinary excretion. Patients with diabetes, especially T2DM, are susceptible to vitamin deficiency due to metabolic disorders such as hyperglycemia that accelerate vitamin consumption; dietary control, such as limiting the intake of fruits and vegetables; and the effects of drugs such as metformin which impair vitamin absorption. Such deficiency may aggravate insulin resistance and increase the risk of neuropathy and cardiovascular disease (CVD) complications.

### 2.2. Folate in Gestational Diabetes Mellitus (GDM)

Gestational diabetes mellitus (GDM) and T2DM have a similar pathogenesis, namely, impaired insulin secretion and aggravated insulin resistance. Women with a history of GDM have a more than 7-fold increased risk of developing T2DM in middle age, and are also at increased risk of hypertension and CVDs. Approximately 35–50% of GDM patients will develop T2DM within 10 years postpartum [6]. In a prospective, multicenter, multiethnic cohort study from the UK involving 4746 patients with T2DM, it was found that the combination of high folate and low vitamin B12 was associated with higher glycemic levels and increased GDM risk [7].

In another cohort study including 40,244 pregnant women, it was found that there was a significant correlation between folate exposure (intake or status) during pregnancy and the risk of GDM, and that folic acid supplementation during pregnancy and childbirth was positively correlated with the risk of GDM [8]. B vitamins and homocysteine (Hcy) may contribute to GDM pathogenesis. In one cross-sectional study, folate, vitamin B6, vitamin B12, and Hcy, in the plasma of 913 pregnant women at 26 weeks of gestation were examined in relation to GDM and glycemic parameters. Higher plasma folate was associated with higher 2 h glucose and higher odds of GDM among Indian mothers. Women with combined vitamin B12 insufficiency and high folate concentrations are most likely to develop GDM. High maternal folate levels and insufficient vitamin B12 are associated with a higher risk of GDM [9]. In a meta-analysis, it was found that red blood cell folate and plasma/serum folate significantly increased the risk of GDM. For every 200 ng/mL increase in red blood cell folate, the risk of GDM increases by 8% [10]. According to a case–control study, either no folic acid supplementation or higher doses of folic acid increased the risk of GDM [11]. Collectively, both insufficient and excessive folate status are detrimental with respect to GDM.

In addition, several studies have indicated that a maternal high-fat and high-sucrose (HFS) diet affects the glucose and lipid balance of offspring, which may also be regulated by folate. A maternal HFS diet leads to increased fasting blood glucose and decreased expression levels of liver metabolism-related enzymes in offspring. Such a diet also alters insulin sensitivity and hepatic lipogenesis in offspring by altering gene and protein expression, and induces insulin resistance. Supplementation with 5 mg/kg folic acid in a maternal HFS diet significantly increased circulating insulin and reduced plasma glucose in offspring [12]. Taken together, existing evidence indicates that an imbalanced status of folate and vitamin B12 is closely linked to elevated GDM risk, while folate also exerts regulatory effects on the long-term glucose metabolism of offspring exposed to a maternal high-energy diet.

### 2.3. Folate in Diabetic Complications

Long-term high blood glucose in T2DM can damage the vascular endothelium and peripheral nerves, leading to multiple diabetic complications including CVDs, diabetic peripheral neuropathy (DPN), retinopathy, peripheral neuropathy, and cognitive impairment. Recent studies have found that folate levels are correlated with the occurrence of diabetic complications.

#### 2.3.1. Folate in CVDs

Hyperglycemia is an important clinical hallmark of T2DM and a major driver of its multiple complications, including CVDs, stroke, and cancer. A 2020 report on cardiovascular health among Chinese T2DM patients revealed that one third of T2DM patients in China suffer from concomitant CVDs, and among patients with cardiovascular complications, more than 90% have atherosclerotic heart disease [13]. Compared with non-diabetics, people with diabetes are 2–4 times more likely to develop CVDs [14]. Low folate concentrations increase the risk of T2DM complications such as CVDs and all-cause mortality [15]. Researchers in a prospective cohort study analyzed 8067 T2DM patients in the National Health and Nutrition Examination Survey (NHANES) from 1999 to 2014 and NHANES III data from 1988 to 1994 to evaluate associations between serum folate and vitamin B12 concentrations and the risk of CVD mortality among T2DM patients. This analysis showed that low serum folate levels in T2DM patients were significantly associated with higher cardiovascular disease mortality [16]. A study performed with restricted cubic-spline (RCS) analysis demonstrated that serum and erythrocyte folate levels were related to cardiovascular and all-cause mortality in hypertensive adults. Furthermore, there was an optimal folate concentration range with respect to CVDs and all-cause mortality [15]. While several studies have verified the association between serum folate levels and CVD risk in at-risk populations, additional interventional research is still required to determine appropriate folic acid supplementation dosages.

Endothelial dysfunction acts as the initiating factor and core pathological basis for the occurrence and progression of CVDs. It promotes vascular injury, thereby inducing and aggravating a variety of cardiovascular disorders. Endothelial dysfunction is also a common feature of T2DM. Folate can improve vascular endothelial function and prevent CVDs. Folate significantly enhances endothelium-dependent vasodilation (FMD) and reduces Hcy levels [17]. Nitric oxide (NO), an important vasodilator released by the vascular endothelium, is synthesized from L-arginine in vascular endothelial cells under the catalysis of endothelial nitric-oxide synthase (eNOS). The main feature of endothelial dysfunction is reduced NO bioavailability or decreased NO synthesis. CVDs in patients with diabetes are characterized by endothelial dysfunction and high cardiovascular mortality. Folate can also prevent eNOS uncoupling and protect against eNOS dysfunction [18].

#### 2.3.2. Folate in DPN

Growing evidence indicates that deficiencies in folate are closely linked to the occurrence of DPN. In a meta-analysis study, the authors summarized and analyzed 16 studies on serum folate levels in patients with T2DM (including 1190 T2DM patients and 1501 controls) and 18 studies on serum vitamin B12 levels (including 1239 T2DM patients and 1562 controls), examining the relationship between serum folate and vitamin B12 levels and the risk of DPN. It was found that compared with T2DM patients, serum folate and vitamin B12 levels in patients with T2DM combined with DPN were further reduced [19]. These findings suggest that supplementation with folate may reduce the risk of developing DPN.

#### 2.3.3. Folate in Cognitive Impairment

Insufficient folate supply is closely associated with cognitive impairment in T2DM. Folate deficiency induces cognitive dysfunction including anxiety-like behavioral disturbances and impaired spatial learning and memory [20]. Folate levels are reduced in T2DM patients and further reduced in T2DM patients with cognitive impairment [21]. Mice fed a chronic folate-deficient diet (CFD) for seven weeks exhibited markedly elevated serum total Hcy. Feeding for 16 weeks induced obesity, lipid metabolism disorders, glucose intolerance, and insulin resistance, whereas feeding for 24 weeks elicited cognitive dysfunction such as anxiety-related activities and impaired spatial learning and memory abilities [20]. Collectively, clinical observations and animal-model data suggest that sustained folate insufficiency may contribute to the progression of diabetes-associated cognitive dysfunction.

#### 2.3.4. Folate in Other Diabetic Complications

Apart from the well-documented CVDs and neurological complications of diabetes, folate status may also interact with endocrine comorbidities in patients with T2DM. T2DM and thyroid dysfunction (TD) are two common chronic endocrine diseases. In a study of 268 patients with T2DM, the authors investigated the correlation between folate deficiency and the risk of elevated thyroid stimulating hormone (TSH). In total, 15.3% of T2DM patients had TD, and 80.5% of T2DM patients had elevated TSH. Serum folate levels were inversely related to TSH; folate deficiency was associated with a higher risk of elevated TSH in patients with T2DM, and serum folate independently predicted elevated TSH risk in this population [22]. This observation suggests that low folate status might be a potential risk factor for thyroid hormone abnormalities among individuals with T2DM.

Beyond the common complications outlined above, the spectrum of diabetic complications encompasses diabetic ketoacidosis (DKA), diabetic nephropathy, cerebrovascular disorders, diabetic foot, infections, skin lesions, and others. Although current research on the association between folate and diabetic complications remains limited, consistent and conclusive clinical and preclinical evidence is lacking, and large-scale cohort studies are still scarce. Nevertheless, in light of available observational data and the pathophysiological mechanisms, it can be reasonably inferred that folate deficiency is as one of the risk factors of diabetic complications.

## 3. Mechanism of Folate Metabolism Involved in Regulating T2DM

Folate metabolism regulates the progression of T2DM through multiple pathways. Folate deficiency leads to Hcy accumulation, triggering oxidative stress and chronic inflammation, which impairs pancreatic β-cell function and exacerbates peripheral insulin resistance. Meanwhile, as a vital methyl donor, folate participates in the epigenetic modification of genes related to glucose and lipid metabolism, regulating insulin signaling pathways at the transcriptional level. In addition, disrupted folate metabolism homeostasis tends to damage endothelial function and further promote the occurrence of CVDs. Nevertheless, the improving effect of folate intervention on glucose metabolism remains to be further explored.

### 3.1. Folate-Mediated One-Carbon Metabolism

**Figure 1:**
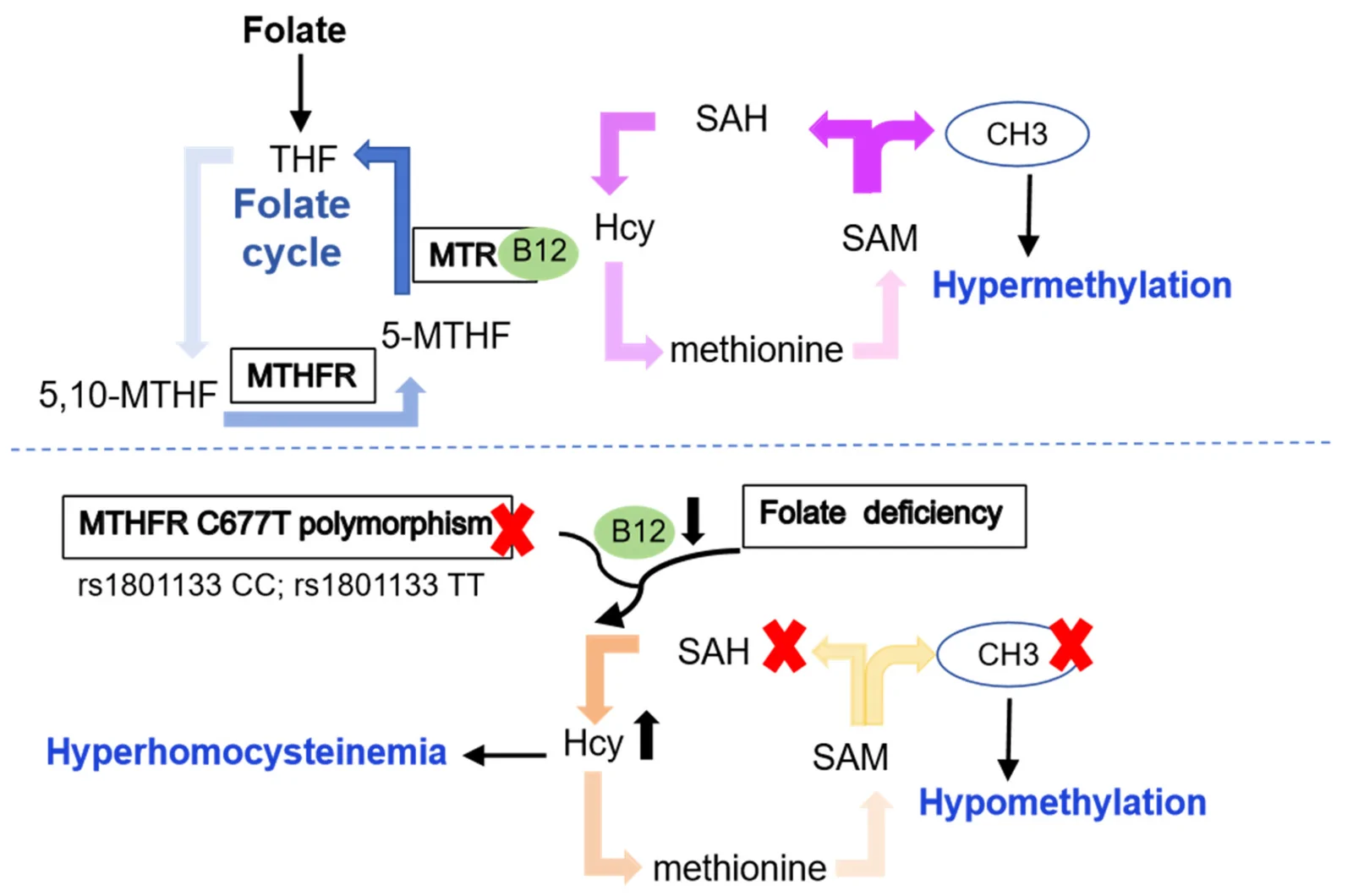
Folate deficiency and MTHFR inactivation cause HHcy and hypomethylation. In the setting of concurrent folate-B12 deficiency, or MTHFR gene mutation, folate metabolism is impaired, leading to the accumulation of Hcy. Disruption of the methionine cycle perturbs SAM-SAH homeostasis, reduces the bioavailability of methyl donors, and ultimately gives rise to hypomethylation.

Folate-mediated one-carbon metabolism (FOCM) plays an important role in various physiological processes including purine and thymidine synthesis, amino acid homeostasis, epigenetics, and redox defense. Disruption of FOCM disturbs physiological homeostasis and normal organismal development. Growing evidence suggests that the dysfunction of the FOCM pathway contributes to the onset and progression of T2DM. Chronic folate deficiency leads to disturbances in glucose and lipid metabolism and subsequent cognitive dysfunction in mice. Compared with non-diabetic patients, patients with T2DM have lower folate levels and higher Hcy levels. In T2DM, impaired FOCM appears to be remodeled via elevated HHcy, oxidative-redox stress, and mitochondrial abnormalities. Accumulating evidence indicates that FOCM-related nutrients such as folate and vitamin B12 are associated with GDM pathogenesis. FOCM-related genetic variation also affects the relationship between folate and vitamin B12 and GDM. High folate and low vitamin B12 concentrations are associated with the development of GDM. The 5,10-methylenetetrahydrofolic acid reductase (MTHFR) locus is located at the end of the short arm of chromosome 1 (1p36.6). MTHFR represents a rate-limiting enzyme in folate metabolism; its enzymatic activity determines cellular folate metabolic capacity. Folate metabolism is an overall process of cellular DNA, RNA, and protein methylation. Pregnant women carrying the MTHFR rs1801131 TT genotype are more likely to develop FOCM nutrition-related GDM [23]. FOCM-related nutrients are also closely related to MTHFR rs1801133 polymorphism and GDM. Serum folate, vitamin B12, and Hcy were measured in 1254 pregnant women, and pregnant women with the MTHFR rs1801133 CC genotype displayed higher serum folate and lower Hcy concentrations. Although the MTHFR rs1801133 genotype was not directly associated with GDM, the MTHFR rs1801133 CC genotype had a significant indirect effect on FPG and GDM risk via folate. Serum folate thus mediates the effects of MTHFR rs1801133 on blood glucose levels and GDM [24]. The MTHFR C677T polymorphism is associated with the occurrence of diabetes. The MTHFR gene mutation causing the C677T polymorphism is located in exon 4, causing valine to be converted to alanine at codon 222, a common variant that reduces the activity of the enzyme [25]. MTHFR genotyping was performed on 236 T2DM patients diagnosed with diabetes, and it was found that people with the MTHFR 677T allele have a 4-fold higher diabetes risk [26]. Individuals with risk factors such as a family history of diabetes or impaired glucose tolerance should be screened for the MTHFR C677T mutation and supplemented with folic acid, which may help delay the onset of diabetes. Patients with the MTHFR rs1801133 TT genotype have a significantly increased risk of ischemic stroke (IS) and coronary artery disease (CAD) [27,28]. Overall, folate deficiency and reduced MTHFR enzyme activity are the primary drivers of impaired folate metabolism, elevated Hcy levels, and disrupted methylation processes (Figure 1). Although high-dose folic acid supplementation can indeed reduce the risk of some diseases caused by MTHFR mutations, for many people with these mutations, folic acid supplementation alone is not enough.

HHcy is considered a risk factor for multiple complications, including cardiovascular and neurological diseases. Folate is an important coenzyme in the Hcy metabolic pathway. After folate enters the human body, it is metabolized into 5-MTHF, which provides a methyl group to Hcy, allowing it to be converted back into methionine. Folate deficiency can affect the activity of MTHFR, further regulate the production of MTHF, and significantly increase Hcy levels. HHcy and hyperglycemia can induce oxidative stress levels in T2DM patients, whereas folic acid supplementation can reduce Hcy and oxidative stress levels. In a randomized controlled clinical trial, researchers studied 68 men with T2DM and found that folic acid supplementation could reduce plasma Hcy and serum malondialdehyde levels and increase serum total antioxidant capacity and folate and vitamin B12 levels in T2DM patients [29]. Plasma Hcy is also a risk factor for vascular disease and a precursor of the antioxidant glutathione. Oral folic acid supplementation for 3 months increased glutathione levels and reduced plasma Hcy in 27 patients with T2DM and microalbuminuria [30]. Hcy triggers a cascade of endocrine disturbances that promote insulin resistance, the main reason for poor glycemic control in most T2DM patients. Mice fed a high-methionine low-folic acid (HMLF) diet developed HHcy accompanied by progressive body-weight loss. Changes in the HMLF diet led to glucose homeostasis and intestinal flora imbalance, resulting in mild glucose intolerance and insulin resistance [31]. Early supplementation of folic acid and vitamin B12 can improve insulin resistance and lipid levels in IUGR rats to a certain extent, while reducing Hcy levels [32]. Therefore, we speculate that folic acid supplementation can reduce Hcy and improve insulin resistance, and may become a potentially effective treatment for patients with T2DM.

### 3.2. Folate Regulates Inflammation and Oxidative Stress

Emerging evidence indicates that folate exerts protective roles in diabetes and its complications largely through modulating inflammatory and oxidative stress responses. Sustained hyperglycemia in patients with diabetes can trigger oxidative stress and inflammatory damage. Folic acid supplementation is therefore expected to mitigate oxidative stress and inflammatory-mediated injury. Patients with T2DM are prone to oxidative stress caused by hyperglycemia. Folic acid supplementation reduces Hsp70 levels in T2DM patients who do not receive insulin treatment, ameliorates oxidative stress, and alleviates T2DM-related injury [33]. Bioactive folate derivatives may also attenuate oxidative stress and inflammation-related damage. In an in vitro model of high glucose-induced oxidative stress and inflammation in bronchial epithelial cells BEAS-2B, the expression of the pro-inflammatory protein NF-κB p50 was significantly reduced in cells supplemented with active folate derivatives. Supplementation with the active folate derivatives 5-MTHF and 10-formyltetrahydrofolic acid (10-FTHF) reverses the damaging effects of high glucose on cells and protects them from the oxidative stress and inflammation induced by high glucose [34]. Periconceptional folic acid supplementation can improve maternal HFD-induced IUGR by reducing placental inflammation and oxidative stress, which may be mediated through the regulation of SIRT1-dependent NF-κB and Nrf2 signaling pathways [35]. Supplementing folic acid has beneficial effects on inflammatory factors and oxidative stress in human promyelomonocytic cells, as it effectively inhibits the inflammatory response of THP-1 cells by inhibiting ROS production and JAK2/STAT3 signaling pathway [36].

Folate also modulates inflammatory responses in complications of T2DM. Inflammation is associated with the risk of mild cognitive impairment (MCI) in T2DM. Serum folate levels are related to MCI and inflammation. Researchers enrolled 126 patients with T2DM (63 patients with and 63 without mild cognitive impairment) to explore whether folate status modifies the association between inflammatory markers and the risk of MCI in this population. It was found that serum pro-inflammatory factors IL-6 and hs-CRP were related to the risk of MCI in T2DM patients, but the significant correlation between hs-CRP and MCI only existed in the low-folate subgroup. Serum folate may thus change the correlation between inflammatory factors and MCI in patients with T2DM [37]. Renal injury is one of the most prevalent complications of diabetes, in which inflammation caused by macrophage infiltration plays an important role. Folic acid protects kidney injury in DN mice by inhibiting M1 macrophage polarization, and this protective mechanism may involve the suppression of inflammatory signaling cascades including the NF-κB pathway [38]. Collectively, folate alleviates hyperglycemia-triggered inflammatory and oxidative stress, and its regulatory effects on inflammation further participate in the progression of multiple diabetic complications.

### 3.3. Folate Regulates Insulin Resistance

Chronic low-grade inflammation and oxidative stress serve as critical pathological mechanisms underlying insulin resistance. Excessive ROS trigger lipid peroxidation and cellular injury, activating inflammatory signaling pathways and inducing abundant pro-inflammatory cytokines. These mediators suppress the phosphorylation of insulin receptor and its downstream targets, block insulin signaling transduction, and reduce insulin sensitivity, thereby contributing to insulin resistance and the progression of T2DM.

In patients with T2DM, the risk of insulin resistance increases with decreasing serum folate levels [39]. Folic acid supplementation may benefit glucose homeostasis and reduce insulin resistance [40]. Early supplementation of folic acid and vitamin B12 improves insulin resistance and lipid levels in IUGR rats to a certain extent [32]. In a cross-sectional study of 1530 non-diabetic adults from the 2011–2012 NHANES a significant inverse association was found between serum folate and insulin resistance [41]. Insulin resistance is one of the causes of GDM, and there is a significant correlation between vitamin B12 and folate levels in late pregnancy and HOMA-IR [42]. GDM can adversely affect the health of the developing fetus. Prenatal folic acid supplementation or maternal folate sufficiency protects offspring from obesity and insulin resistance. Maternal folic acid may potentially protect against obesity/insulin resistance in animal models and human offspring of pregnant women [43]. Low vitamin B12 and high folate levels during pregnancy are associated with visceral obesity and insulin resistance in offspring. HOMA-IR, red blood cell folate, Hcy, and methylmalonic acid were prospectively evaluated in 278 consecutive obese patients. Red blood cells, folate, Hcy, and MMA were found to predict HOMA-IR in severely obese patients [44]. Maternal Hcy concentrations are positively correlated with children’s glucose concentrations. The higher the mother’s folate concentration, the higher the children’s HOMA-IR. Maternal Hcy and folate concentrations are significantly associated with birth weight, childhood insulin resistance, and offspring hyperglycemia [45]. This also shows that folate mediates one-carbon metabolism (OCM) disorders and plays an important role in diabetic fetal programming.

Collectively, folate status exerts multifaceted effects on insulin resistance in both adults and their offspring, yet its regulatory effect appears to be context-dependent.

### 3.4. Folate and DNA Methylation

Epigenetics refers to heritable changes in gene expression in the absence of alterations to the DNA sequence and is thought to represent a mechanism linking environmental influences to the etiology of T2DM. As a core methyl donor of OCM, folate participates in the epigenetic regulation of glucose metabolism and diabetic lesions by mediating DNA methylation modification, which is a crucial epigenetic mechanism linking metabolic homeostasis and T2DM pathogenesis. The diabetic state upregulates the activity and abundance of glycine N-methyltransferase (GNMT), a key protein that regulates folate, methyl, and Hcy metabolism. The one-carbon unit derived from folate is the main source of methyl groups, and 5-MTHF allosterically inhibits GNMT. The activities of GNMT, phosphatidylethanolamine N-methyltransferase (PEMT), and betaine-homocysteine S-methyltransferase (BHMT) in the liver of diabetic rats increased approximately 2-fold, and folate deficiency led to a further elevation in GNMT activity. The high glucose state of diabetes disturbs methyl, choline, and Hcy metabolism, and folic acid supplementation can reverse these metabolic alterations to a certain extent [46]. Maternal nutrition and dietary supplements in the maternal diet, such as folic acid, may affect the DNA methylation of the offspring and thus their biological characteristics, thereby modifying their biological phenotypes. Folate may also play a role in T2DM susceptibility by regulating changes in DNA methylation patterns of genes involved in glucose metabolism. Changes in hepatic lipid metabolism and skeletal muscle glucose uptake in offspring whose mothers were fed a HFS diet, as well as insulin resistance in HFS/folic acid supplementation (HFS/F) offspring, may be related to DNA methylation of genes related to insulin secretion [12]. The liver plays an important role in maintaining the body’s glucose metabolism homeostasis. In the setting of insulin resistance among patients with T2DM, the rate of hepatic gluconeogenesis increases, further leading to elevated fasting blood glucose. Compared with non-diabetic subjects, diabetic patients showed differences in DNA methylation, with 251 CpG sites in the livers of T2DM patients. These include genes related to the development of T2DM, such as GRB10, ABCC3, MOGAT1, and PRDM16. The vast majority of important CpG sites (94%) in the livers of T2DM patients displayed reduced DNA methylation. Reduced circulating folate levels may partly explain hepatic hypomethylation observed in individuals with diabetes [47]. Calcium/calmodulin-dependent protein kinase 2 (CAMKK2) is involved in the regulation of glucose homeostasis processes. Folate deficiency is associated with lower CAMKK2 methylation, and CAMKK2 methylation is inversely correlated with the homeostatic model assessment for insulin resistance (HOMA-IR) index. There is thus a direct correlation between low folic acid intake, reduced methylation of the CAMKK2 gene, and insulin resistance in obese individuals [48]. Folate-mediated DNA methylation modification also plays an important role in the occurrence of T2DM-related complications. Patients with T2DM have a high incidence of Alzheimer’s disease (AD). β-Amyloid aggregation and tau hyperphosphorylation are pathological hallmarks of AD. Tau-protein hyperphosphorylation has been detected in both diabetic animals and human patients with diabetes. Folate can reduce tau phosphorylation by regulating protein phosphatase 2A (PP2A) methylation in diabetic mice [49]. Therefore, folate may serve as a promising therapeutic target for diabetes-related cognitive dysfunction. Folate-mediated methylation of OCM genes is also involved in regulating the occurrence and development of T2DM. By detecting the methylation of allelic CpG-SNP sites in T2DM patients, it was found that the OCM pathway gene MTHFD1 rs2236225 in the folate pathway is significantly related to T2DM. The SNP rs2236225 at the MTHFD1 CpG site is methylation-dependent, and regulating the methylation level at this site may interfere with the OCM pathway in T2DM patients [50]. MTHFR mediates the prominent protective effects of folate in an STZ-induced mouse model of diabetic cardiac fibrosis. Promoter methylation of DNMT3A and MTHFR genes was increased in cardiac tissues from patients and mice with diabetic cardiac fibrosis, accompanied by down-regulated MTHFR expression. Knocking down DNMT3A demethylated the MTHFR promoter, restored MTHFR levels, and alleviated diabetic cardiac fibrosis as well as cardiac fibroblast pyroptosis. Folic acid supplementation can rescue the effects of MTHFR loss in diabetic cardiac fibrosis, indicating that epigenetic silencing of MTHFR by folate-dependent DNA methylation is a key mediator of diabetic cardiac fibrosis [51].

Overall, folate status dynamically modulates the DNA methylation of glucose metabolism-related genes and OCM genes, thereby participating in the entire continuum of T2DM onset and progression and multiple diabetic complications. Additionally, folate modulates cellular energy metabolism in the setting of hyperglycemia, which contributes to the amelioration of insulin resistance. Primary human skeletal muscle cells (HSkMCs) were cultured under high-glucose conditions to simulate diabetes. HSkMCs with clinically low folic acid levels gradually underwent morphological changes, insulin resistance, and changes in energy metabolism, manifested by reduced lactate release and pyruvate dehydrogenase E1 alpha (PDHA) expression; the ratios of NADP/NADPH and NAD+/NADH were reduced, and characteristics of late T2DM appeared, indicating that metabolic folic acid stress can promote metabolic memory to convert bioenergy flux and metabolic flexibility, thereby regulating T2DM [52]. In summary, folate can ameliorate insulin resistance via multiple pathways including OCM, DNA methylation, inflammatory response, oxidative stress, and energy metabolism, thereby delaying the onset and progression of T2DM and its complications.

## 4. Therapeutic Effect of Folate on T2DM

### 4.1. Folic Acid Supplementation on Glycemic Homeostasis and Insulin Resistance in T2DM

A meta-analysis including 21,081 patients with diabetes across 18 studies revealed that folic acid supplementation (0.15~15.00 mg/d) may reduce fasting blood glucose (FBG), insulin resistance, and insulin levels, suggesting that folic acid may have potential benefits in improving insulin resistance and glycemic control [53]. A meta-analysis included 29 randomized controlled trials (RCTs) with a total of 22,250 patients, 11 of these RCTs involved 834 patients with diabetes. It was reported that folic acid supplementation alone or in combination with other B vitamins can reduce fasting insulin levels [40]. The association between dietary folate intake and diabetes risk has been explored in several prospective observational studies. A Japanese prospective study involving 19,168 healthy individuals indicated that higher dietary intakes of vitamin C, vitamin B2, and folate were associated with a lower risk of diabetes in women. Thus, greater dietary consumption of vitamin C, vitamin B2, and folate may decrease the risk of T2DM in Japanese women [54]. The authors of a US prospective study followed 4704 diabetes-free adults aged 18–30 and found that folic acid intake was negatively correlated with the incidence of diabetes; moreover, vitamin B6 or vitamin B12 intake was negatively correlated with the incidence of diabetes. Higher folic acid intake corresponded to lower plasma Hcy, lower insulin, and reduced serum C-reactive protein (CRP). Long-term follow-up further suggested that folic acid intake in early adulthood predicted lower diabetes risk in middle-aged US adults [55]. Collectively, these studies support that folate levels in the blood of patients with T2DM are reduced, and that intake of folic acid may mitigate the risk of T2DM. Nevertheless, conflicting observational findings exist. Researchers in a cross-sectional study examined the relationship between dietary folate and niacin intake and diabetes risk in Chinese adults. Diabetic patients had lower vitamin intake, especially lower intake of B vitamins. Diabetic patients also had significantly higher median folate levels than controls, while their median niacin levels were significantly lower. Dietary folic acid and niacin intakes were thus independently associated with the risk of diabetes, with folic acid supplementation increasing that risk, while niacin intake reduces it [56]. Metformin, the first-line agent for T2DM, improves the body weight of diabetic patients and alleviates fatty liver disease, yet long-term metformin treatment substantially impairs folate and vitamin B12 absorption, meaning that regular folic acid and vitamin B12 supplementation may be required for metformin-treated patients with T2DM [57]. Collectively, available observational data yield inconsistent conclusions regarding circulating folate status, dietary folate exposure, and T2DM risk, partly because many datasets derive from observational rather than large-scale randomized controlled trials.

### 4.2. Targeting Folate–Homocysteine Axis and FOCM in T2DM

Folate metabolic processes play an important role in nucleic acid synthesis, amino acid homeostasis, epigenetic maintenance, redox defense, and methylation. Methionine interacts with ATP to synthesize SAM under the catalysis of methionine adenosyltransferase. Catalyzed by methyltransferase, SAM is methylated by transferring a methyl group to another substance, and SAM is changed to SAH, which is dealkylated to produce Hcy. This metabolic pathway requires folate as a methyl group donor to restore methionine. Acute and long-term exposure to high Hcy concentrations adversely affects cell viability and function of β cells [58,59]. It is widely accepted that serum folate concentrations are significantly decreased in patients with T2DM, which has generated the hypothesis that folic acid supplementation may exert therapeutic effects against T2DM. Consistent with this idea, T2DM patients with distinct Hcy levels present significant differences in uric acid, creatinine, urinary microalbumin, urinary albumin-to-creatinine ratio, and nerve conduction velocity. Assessing blood Hcy levels in T2DM patients can help prevent microvascular complications. High Hcy is independently associated with the incidence of diabetic neuropathy in T2DM patients. Compared with T2DM patients without DPN, T2DM patients with DPN have lower serum folate levels [60]. Reducing Hcy levels emerges as a promising treatment for diabetic neuropathy and microvasculopathy. Folic acid supplementation in T2DM patients can reduce Hcy levels and improve glycemic control [61]. A prospective study was conducted on 7333 Korean adults aged 40 years or older to explore the correlation between dietary folic acid intake and the risk of T2DM. Dietary folic acid intake was inversely related to the risk of T2DM in women, but not in men [62]. A double-blind randomized controlled clinical trial was conducted on 100 T2DM patients. Folic acid supplementation (FAS) may help reduce Hcy levels and improve insulin resistance in T2DM patients [63]. The authors of a meta-analysis comprehensively summarized reports in the literature and found that folic acid supplementation reduced fasting insulin and HOMA-IR values, but had no overall effect on fasting blood glucose or HbA1c. This shows that folic acid supplementation may be beneficial to glucose homeostasis and reduce insulin resistance [40].

However, some studies have reported that folic acid supplementation may not alleviate the progression of T2DM. Homocysteinemia may play an etiological role in the pathogenesis of T2DM by promoting oxidative stress, systemic inflammation, and endothelial dysfunction. However, lowering Hcy levels through daily supplementation of folic acid, vitamins B6 and B12 does not reduce the risk of T2DM in women at high risk of CVDs [64]. Mild HHcy is a cardiovascular risk factor for T2DM. Hcy may exert its deleterious effects by inducing endothelial dysfunction and/or chronic inflammation. In T2DM patients with mild hyperhomocysteinemia, lowering Hcy with folic acid for 6 months did not improve biochemical markers of endothelial dysfunction or low-grade inflammation [65]. Therefore, whether folic acid supplementation to reduce Hcy can be used as a treatment for T2DM and to reduce its complications still requires more clinical research results with a larger sample size as a basis for treatment.

Folate enters the body and is catalyzed by MTHFR to generate 5-MTHF. As the main methyl donor in the body, Hcy is methylated into methionine to generate the methyl donor SAM, which participates in the biological process of DNA methylation. Insufficient intake of folic acid, MTHFR gene mutation, vitamin B12 deficiency, etc., can cause folate metabolism disorders, leading to HHcy. Studies have reported that high levels of Hcy are one of the main causes of insulin resistance. Insulin receptors are located on the cell membrane and are responsible for receiving insulin stimulation and initiating insulin signal transmission. High levels of Hcy spontaneously modify the cysteine site of the insulin receptor precursor protein through disulfide bonds, disrupting the maturation process of the insulin receptor in the endoplasmic reticulum and Golgi apparatus, ultimately leading to a significant reduction in the mature form of the insulin receptor protein, causing the transmission of insulin signals to be directly inhibited in the first step, and leading to severe insulin resistance [66]. Therefore, it is speculated that reducing Hcy by supplementing folic acid can effectively inhibit insulin resistance. In addition, although most studies report that MTHFR gene mutations are closely related to insulin resistance in T2DM patients, no effective treatment has been found to treat this gene defect. However, it is usually recommended that patients with MTHFR gene mutations supplement a large amount of folic acid to compensate for the impact of the lack of MTHFR enzyme activity. However, this treatment is still experimental, and the standard of treatment and its effectiveness are still questionable.

### 4.3. Folic Acid Supplementation Improves T2DM-Related Complications

#### 4.3.1. CVDs

CVDs, including coronary artery disease (CAD), cerebrovascular disease, heart failure, and peripheral arterial disease (PAD), are one of the most common complications in patients with T2DM. In patients with T2DM (including those with microvascular complications), atherosclerosis progresses more rapidly, often leading to premature CVD events. Among adults in the U.S. NHANES from 1988 to 1994, patients with T2DM and CVDs had the highest mortality [67]. Therefore, it is particularly important to find effective drugs and therapeutic targets to improve T2DM and T2DM-related cardiovascular diseases. In a prospective cohort study of 9266, 12,601, and 16,025 U.S. adults with diabetes, prediabetes, and insulin resistance (homeostatic model assessment of IR >2.6) from the NHANES III and 1999–2018, respectively, increasing daily folic acid intake may help reduce all-cause and cardiovascular mortality in adults with dysglycemia [68]. The 4977 bp mitochondrial deletion (mtDNA4977 deletion) is a marker of mitochondrial oxidative damage and may play an important role in CAD. The authors of a cross-sectional analysis of angiographic data in diabetic patients who did not supplement B vitamins in 2017 found that the interaction between mtDNA4977 deletion in leukocytes and folate deficiency in diabetic patients may significantly increase the risk of obstructive CAD [69]. A hospital-based case–control study in China, including 419 T2DM patients with newly diagnosed CVD and 419 age (±5 years) and gender-matched T2DM controls, high folic acid and vitamin B6 intake may be associated with lower risk of CVDs in patients with T2DM. It is suggested that dietary folic acid and vitamin B6 have a protective effect on cardiovascular disease in patients with T2DM [70]. In a cross-sectional analysis of 6633 overweight/obese participants with MetS (metabolic syndrome) among older adults at higher cardiometabolic risk, increased folic acid intake was associated with lower MetS scores and was positively correlated with HDL cholesterol, indicating that higher folic acid intake may be associated with lower MetS scores in older adults, lower plasma fasting plasma glucose, and higher HDL cholesterol in high-risk cardiometabolic subjects [71]. The authors of another study included survival data of 9196 T2DM patients and analyzed the relationship between folic acid intake and mortality risk. It was found that higher dietary intake of folic acid was significantly associated with lower all-cause mortality and cardiovascular mortality. Increasing dietary folic acid intake may thus reduce the risk of death in adults with T2DM [72].

Diabetes is associated with endothelial dysfunction, which may be related, in part, to the uncoupling of endothelial nitric oxide synthases (NOS), thereby reducing NO availability. Because folic acid may potentially reverse the uncoupling of NOS, folic acid supplementation significantly increased folate levels and decreased plasma Hcy levels in a randomized, placebo-controlled, crossover study. Folic acid supplementation for 2 weeks can thus improve endothelial dysfunction in T2DM patients [17]. Sustained hyperglycemia in diabetic patients leads to increased vascular superoxide production, which inactivates NO and causes vascular dysfunction. Folate, on the other hand, improves endothelial cells in diabetic patients, and preserves NO bioactivity due to its Hcy-lowering effects, antioxidant effects, and effects on cofactor availability. Folic acid supplementation may improve delayed diabetic wound healing by increasing NO bioavailability [73]. Folic acid intake also ameliorated acetylcholine-induced aortic vascular blunting relaxation in obese/diabetic mice. The mechanism may be related to the enhancement of the PI3K/HSP90/eNOS/Akt cascade, reduction in plasma resistin levels, downregulation of PTEN and slight changes in oxidative status [74]. Hyperglycemia causes oxygen radical stress but is also associated with the uncoupling of endothelial NOS. The B vitamin folate has a direct beneficial effect on endothelial function. Folate plasma concentration determined endothelium-mediated vasodilation in T2DM patients [75]. Injections of the active form of folic acid, 5-MTHF, improved NO-mediated vasodilation in patients with T2DM [76]. This suggests that folate can be used to improve NO bioavailability and restore endothelial dysfunction in T2DM.

#### 4.3.2. Folic Acid Supplementation Improves Other Complications of T2DM

Researchers study analyzed the therapeutic effect of folate on diabetic retinopathy (DR) in obese T2DM genetic model mice. Folate plays a protective role in retinal thinning in the early stage of DR in db/db mice. The levels of molecules related to angiogenesis, inflammation and oxidative stress in the retina and serum of folic acid-treated diabetic mice were significantly down-regulated, and serum Hcy levels were also significantly reduced. Folate may thus serve as a potential treatment for DR by inhibiting angiogenesis, inflammation, and oxidative stress [77]. Depression is also associated with a higher risk of new-onset T2DM. The use of SSRI antidepressants may increase the risk of new-onset T2DM by causing oxidative stress in pancreatic cells. Fluoxetine-induced defects in β-cell function can be prevented by adding the antioxidant folic acid. Folic acid supplementation in patients taking SSRIs may reduce the risk of new-onset diabetes by protecting normal beta cell function [78]. Diabetes is a metabolic disorder that leads to serious complications. Supplementing folic acid can alleviate the increase in alkaline phosphatase, decrease albumin and fibrinogen concentrations, reduce MMP-2 activity, and increase urea and creatinine concentrations [79]. Dietary folic acid supplementation can also reduce streptozotocin-induced cardiomyocyte apoptosis in diabetic patients [80]. It can be seen that folate also has a protective effect on the heart and liver in diabetic rats. In addition, folic acid intake has a significant regulatory effect on β-adrenergic receptor protein expression and lipolysis in the adipocytes of obese/diabetic mice [81]. Metabolic folate stress can promote metabolic memory to transform bioenergetic flux and metabolic flexibility, thereby regulating late-stage T2DM [52].

Folate also mediates T2DM disease through other mechanisms. For example, T2DM is associated with an increased frequency of DNA damage. Folate deficiency renders cells susceptible to glucose-induced DNA damage. To investigate whether the B lymphoblastoid WIL2-NS cell line cultured under folate-deficient conditions is more sensitive to glucose-induced DNA damage, WIL2-NS cultured under folate-deficient conditions was found to have an increased frequency of DNA damage when exposed to high concentrations of glucose. T2DM patients with hyperglycemia and folate deficiency may be at higher risk of chromosomal instability [82]. As T2DM progresses, sugar auto-oxidation is promoted, leading to the generation of reactive oxygen species. This damage occurs particularly at the level of cellular proteins, carbohydrates, lipids, and DNA. T2DM oxidative stress is positively correlated with DNA damage, and folic acid supplementation can reduce the number of micronuclei, 8-OHdG, and lipid peroxide content in T2DM patients. Therefore, supplementing folic acid in the treatment of T2DM may help delay complications caused by oxidative stress and DNA damage in T2DM [83]. Metformin is the first-line treatment for patients with T2DM and GDM to maintain glycemic control. Metformin is associated with maternal vitamin B12 deficiency and anti-folic acid activity. The balance of vitamin B12 and folate is crucial for OCM. An imbalance of folate and vitamin B12 may lead to damage to the process of DNA methylation and nucleic acid purine/pyrimidine synthesis, further causing genome instability and abnormal gene expression. Imbalances in folate and vitamin B12 can also affect mitochondrial aerobic respiration, thereby inhibiting placental and fetal growth, as well as the activity of mammalian target of rapamycin (mTOR) cellular nutrient transport [84].

## 5. Conclusions and Limitations

Existing studies have confirmed that folate is closely related to T2DM, GDM, and diabetic complications. Imbalanced folate levels constitute an important risk factor for the development of glucose metabolism disorders and insulin resistance. On the one hand, most patients with T2DM have low serum folate levels, which can be attributed to increased glucose consumption, metformin-induced inhibition of folate absorption, and elevated urinary excretion. Although reduced serum folate is widely regarded as a common characteristic in diabetic patients, some studies have reported contradictory findings. For instance, researchers compared fasting serum folate levels in male subjects with normal glucose tolerance (NGT), impaired glucose tolerance (IGT), newly diagnosed T2DM, and previously diagnosed T2DM, and found that serum folate levels were significantly higher in both newly and previously diagnosed diabetic patients than in the NGT group [85]. Another study further demonstrated that high red blood cell folate concentrations were significantly associated with an increased risk of all-cause mortality in adults with diabetes [86]. These inconsistent results indicate that in the T2DM population, factors other than serum folate levels are also implicated in the pathogenesis and prognosis of the disease.

**Figure 2:**
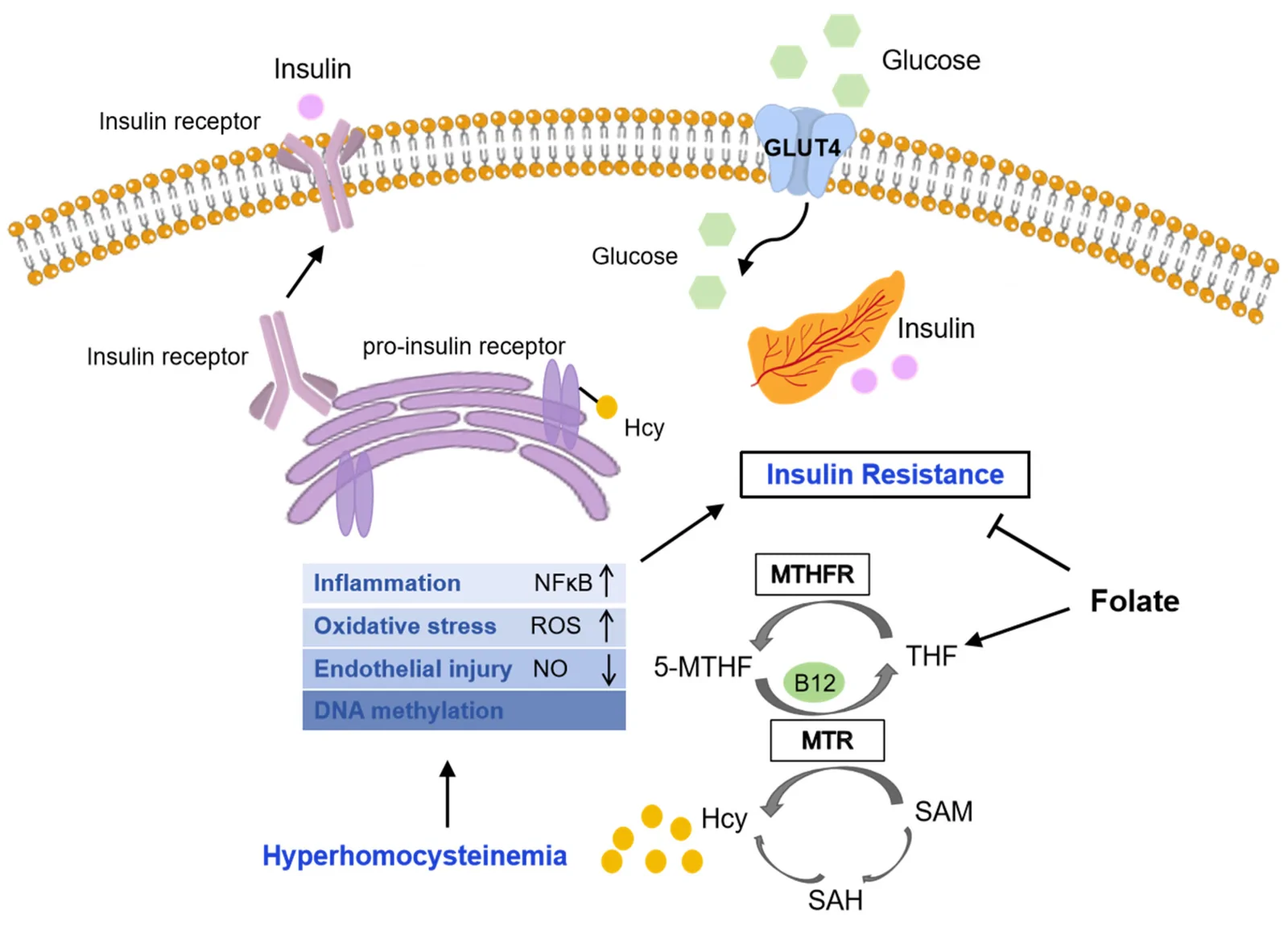
Mechanisms of folate and HHcy in insulin resistance. Folate deficiency leads to hyperhomocysteinemia contributes to insulin resistance via multiple interconnected pathways. Elevated Hcy triggers endothelial injury, oxidative stress, inflammation, and aberrant DNA methylation.

Folate deficiency is often accompanied by HHcy, which exacerbates oxidative stress and chronic inflammation, thereby causing damage to the vascular endothelium, peripheral nerves, kidneys, and myocardium (Figure 2). Appropriate folic acid supplementation can improve insulin resistance and reduce the risk of diabetic complications through multiple mechanisms, including regulating FOCM, lowering Hcy levels, inhibiting inflammatory responses, and restoring eNOS function. On the other hand, the biological effects of folate are dose-dependent, and inappropriate intake may lead to metabolic imbalance, especially in pregnant women. Isolated high folate intake combined with vitamin B12 deficiency significantly increases the risk of GDM, and excessive folic acid supplementation may also predispose certain individuals to long-term adverse metabolic outcomes. Collectively, these findings suggest that folate intervention should be maintained within an optimal dose range, and its efficacy is closely related to the status of other B vitamins, particularly vitamin B12. Notably, the therapeutic effects of folic acid do not exhibit a simple dose-dependent relationship despite broad dose variations across studies [53]. A 5.00 mg/d threshold is identified for significant improvements in HOMA-IR and FBG. Predominantly folate-insufficient study populations may alter treatment responses. High-dose folic acid (5.00~15.00 mg/d) carries risks of masking vitamin B12 deficiency in patients with diabetes. Given the short-term nature of available trials, long-term dose–response and safety profiles of high-dose folic acid supplementation remain unclear, necessitating further long-term RCTs.

Nevertheless, current evidence is insufficient to develop standardized folate intervention protocols for diabetic patients, identifying four key research priorities. First, large scale, genetically stratified RCTs are essential to screen T2DM subgroups responsive to folate supplementation. MTHFR polymorphisms can clarify individual treatment responses and prevent ineffective or hazardous supplementation in non-responders. Second, the optimal folate preparation and dose require definition. Third, the translational gap between rodent mechanistic data and clinical practice necessitates more human-based mechanistic studies due to interspecies physiological differences. Fourth, the interplay between metformin and folate/B12 status needs further exploration.

Promising pre-clinical and mechanistic data have not translated into clinical benefits of folate for T2DM and its complications. Folate-mediated Hcy reduction failed to lower T2DM incidence in high-cardiovascular-risk women [64], and six-month folate-based therapy produced no improvement in endothelial or inflammatory biomarkers among T2DM patients with mild HHcy [65]. Such inconsistencies arise from multiple limitations: reliance on observational cohorts vulnerable to confounding and reverse causation, and population heterogeneity exemplified by sex specific associations in Asian cohorts [62]. Thus, folate-dependent lowering Hcy cannot yet be confirmed as a definitive therapy for T2DM and its complications. Further prospective RCTs stratified by genotype, sex and baseline B vitamin status are urgently needed for clinical validation.

## Data Availability

No new data were created or analyzed in this study. Data sharing is not applicable to this article.
